# Dr. Christian Fenger

**Published:** 1902-04

**Authors:** 


Dr. Christian Fenger, of Chicago, died at his home in that city March 7, 1902, of croupous pneumonia, at the age of 61 years. He was a surgeon of eminence and noted for his scientific attainments.

